# Pathogenic bacteria enriched in the oral microbiota might be associated with recurrent pulmonary infections in elderly individuals

**DOI:** 10.1007/s40520-025-03141-1

**Published:** 2025-08-13

**Authors:** Jingyi Xu, Ruyi Qu, Keke Yang, Yuezhu Wang, Meiyun Nie, Xiaodong Qi, Huajun Zheng, Ling Yang

**Affiliations:** 1 Department of Geriatrics, Shanghai Fourth People’s Hospital Affiliated to Tongji University, Shanghai, 200434 China; 2 Shanghai-MOST Key Laboratory of Health and Disease Genomics, NHC Key Lab of Reproduction Regulation, Shanghai Institute for Biomedical and Pharmaceutical Technologies, Shanghai, 200237 China

**Keywords:** 16S rRNA gene, Oral microbiota, Gut microbiota, Recurrent pulmonary infections, Elderly individuals

## Abstract

**Background and aims:**

Pulmonary infections are a major health concern for the elderly, because of their high morbidity and mortality rates. With the growing world’s aging population, it is crucial to prioritize the health of elderly individuals. This study aimed to explore the associations between oral and gut microbiota and pulmonary infections.

**Methods:**

Throat swabs and stool samples were collected from elderly patients aged 78–98 years and divided into four groups: Control, Infection, Re-Infection, and Re-None. The microbiota were analyzed via 16S rRNA gene sequencing, and the functional predictions were imputed using PICRUSt with MetaCyc pathway annotation.

**Results:**

Significant differences were observed in oral and gut microbiota diversity between the control and test groups. Patients with pneumonia showed a significant increase in *Staphylococcus aureus* abundance in the oral microbiota compared to the Control group, while those with recurrent pneumonia showed elevated, *Klebsiella pneumoniae* levels. In the gut microbiota, *Enterococcus hirae* alone that was significantly enriched in all three test groups. Furthermore, PICRUSt2 analysis indicated an increased relative abundance of genes associated with the degradation of D-glucarate and D-galactarate pathways in patients with recurrent infections.

**Conclusion:**

Oral and gut microbiota diversity showed significant differences between patients with recurrent pneumonia and common pneumonia pneumonia-infected patients. The higher prevalence of both *S. aureus* and *K. pneumoniae* in the oral microbiota offers crucial insights into the pneumonia etiology. Specifically, the increased abundance of *K. pneumoniae* may contribute significantly to the heightened lung infections susceptibility among elderly individuals.

**Supplementary Information:**

The online version contains supplementary material available at 10.1007/s40520-025-03141-1.

## Background

Recurrent pulmonary infections represent a significant health concern among elderly individuals due to increased risk of morbidity and mortality, contributing to hospital admissions and healthcare costs [1]. Elderly individuals are more susceptible to pulmonary infections because of age-related decline in lung defenses and, immunity, along with added comorbidities, and inadequate oral hygiene [2]. Clinical presentation of infections such as pneumonia in the elderly can be particularly difficult owing to the presence of pre-existing pneumonia-like cardiopulmonary disease [3]. Due to hospitalization costs and long-term outcomes, pneumonia places a significant burden on health care resources, specifically in elderly patients [4]. Given the dire situation, the elderly patients with pneumonia deserve particular attention.

Oral microbiota plays a role in maintaining lung homeostasis [5], with the oral cavity hosting the second most diverse microbial community in humans. This community encompasses over 700 bacterial species, alongside microeukaryotes, archaea, and viruses. The oral microbiome is correlated with systemic diseases such as pulmonary and, cardiovascular diseases, rheumatoid arthritis, and respiratory illnesses [6, 7]. Notably, *Staphylococcus aureus* and *Klebsiella pneumoniae* are the two bacteria most frequently implicated in pulmonary infections [7, 8].

*Klebsiella pneumoniae* is a Gram-negative, facultative anaerobe, recognized as an opportunistic pathogen capable of causing severe pneumonia, particularly in immunocompromised individuals [9]. The capsule polysaccharide of *K. pneumoniae* is a well-known virulence factor that promotes resistance to phagocytosis and serum bactericidal activity [10]. The horizontal transfer of multiple antibiotic resistance genes through plasmids, transposons, and genetic elements, *K. pneumoniae* difficult to treat [11].

*Staphylococcus aureus*, a Gram-positive opportunistic pathogen is responsible for a broad spectrum of clinical infections, ranging from moderately severe skin infections to fatal pneumonia and sepsis [12, 13]. *S. aureus* evades the host immune response and antibiotic treatment through a multitude of distinct mechanisms, such as blocking leukocytes chemotaxis, sequestering host antibodies, preventing detection via polysaccharide capsules or biofilm formation, and resisting degradation after phagocyte ingestion [13]. *S. aureus* can secrete a variety of enterotoxins and other toxins to trigger inflammatory responses and activate immune cells [14].

Gut microbiota, an integral part of the human body, the gut microbiota has evolved alongside its host [15] and is closely related to maintaining host health. It has been implicated in conditions such as inflammatory bowel disease, major depressive disorder, and schizophrenia, and even regulates pathogens and pathobionts [16, 17]. It can also influence the lung immune system through both local and systemic interactions [18]. In particular, gut microbiota has been shown to regulate immune responses at distal mucosal sites, especially in the lungs [19]. Gut microbiota dysbiosis has been linked to several lung diseases, such as chronic obstructive pulmonary disease, asthma, and Staphylococcal pneumonia [20].

Microbiota composition and diversity is commonly analyzed using 16S rRNA gene sequencing [21]. The 16S rRNA gene is a fragment of the prokaryotic the ribosomal small subunit, and it contains both conserved region and variable regions(V1–V9) [22]. The variable regions are used for species discrimination. Typically, the V3–V4 or V4–V5 variable regions are amplified via polymerase chain reaction (PCR) and analyzed using high-throughput sequencing to determine the microbiota composition.

In this study, we observed differing frequencies of pneumonia infections among the elderly patients (average age, 92 years) with long-term hospitalization. While occasional infection in elderly patients is common due to age-related vulnerabilities [1, 4], the primary cause underlying frequent infections among hospitalized individuals merits further study. The potential association between recurrent pulmonary infections and oral microbiota structure, especially the presence of pathogens such as *K. pneumoniae* and *S. aureus*, must be investigated. To develop effective prevention and treatment strategies for recurrent pulmonary infections, understanding the distribution of these bacteria in the oral cavity and their impact on the elderly population is crucial. Therefore, this study aimed to explore the connection between the oral microbiota and recurrent pneumonia in elderly individuals. Gut microbiota was also analyzed to identify potential correlations with the oral microbiota. By elucidating the underlying risk factor mechanisms and, predicting microbiota function, this study provides clinical evidence and, valuable insights that may guide healthcare improvements for elderly individuals.

## Methods

### Sample collection

The study was conducted on elderly patients at Shanghai Fourth People’s Hospital affiliated with Tongji University. The characteristics, clinical features, and laboratory data of the patients were obtained, and throat and stool samples were collected at three separate time points: 26 October 2023, 27 December 2023, and 1 March 2024.

The initial number of patients included 1,524. Through intense screening, the following patients were excluded: 72 with tumors, 10 with severe liver and kidney damage, 40 with cardiovascular and cerebrovascular accidents, 32 with critical conditions, 52 with non-pulmonary infections, and 1,256 without long-term hospitalization. Long-term hospitalization was defined as continuous stay in a hospital or nursing home. Finally, 62 participants with an average age of 92 years were enrolled in the study. Based on the patients’ clinical status at the time of sampling, they were divided into four groups: the Control, Infection, Re-Infection and Re-None (Table 1). Recurrent pneumonia was defined as > 5 episodes of pneumonia infection within a year. The groups were defined as follows: Control group, < 5 episodes of pneumonia infection in the preceding year with no current infection at the time of sampling (Supplementary Fig. S1); Infection group, < 5 episodes of pneumonia infection in the preceding year but infected at the time of sampling; Re-Infection group, recurrent pneumonia and infected at the time of sampling; Re-None group, recurrent pneumonia but no current infection at the time of sampling.

The following data were collected: age, sex, C-reactive protein level, neutrophil count, leukocyte count, and other relevant clinical indices. Throat swabs and stool samples were collected from each patient to analyze the oral and gut microbiota. Samples were collected thrice independently at each time point. Importantly, as patients’ physiological or pathological states may vary between sampling time points, identical grouping of samples across all collections could not be guaranteed.

**Table 1:** Population characteristics of samples

		Re-Infection	Re-None	Infection	Control	Total
Grouping situation	Recurrent lung infections	yes	yes	no	no	
	Infection at the time of sampling	yes	no	yes	no	
First sampling (Date:2023-10-26)	Person-times of Sampling	8	11	4	20	43
	Sampling from gut	8	11	4	20	43
	Sampling from throat swab	8	10	4	20	42
	The average age of patients	93.2(90–96)	91.38(82–95)	94.33(91–97)	91.38(82–95)	
Second sampling (Date:2023-12-27)	Person-times of Sampling	5	12	4	23	44
	Sampling from gut	5	12	4	22	43
	Sampling from throat swab	4	11	4	21	40
	The average age of patients	92.63(90–95)	91.38(82–95)	93.66(91–96)	92.56(83–96)	
Third sampling (Date:2024-03-01)	Person-times of Sampling	7	5	5	21	38
	Sampling from gut	7	4	4	20	35
	Sampling from throat swab	6	4	4	17	31
	The average age of patients	94.00(93–95)	92.38(82–95)	93.86(91–97)	91.53(78–98)	
Total	Person-times of Sampling	21	28	13	64	126
	Sampling from gut	20	28	12	62	122
	Sampling from throat swab	18	25	11	58	112
	The average age of patients	93.33(90–96)	92.41(82–95)	94.0(91–97)	92.44(78–98)	

### Genomic DNA extraction, PCR amplification, and gene sequencing

The genomic DNA was extracted from stool using the MagicPure 32 Stool and Soil Genomic DNA Kit (TransGen, China) and from mouth swabs using the TIANamp Swab DNA Kit (TIANGEN, China). The V3-V4 region of the 16S rRNA gene was amplified using the primers pair 338F (ACTCCTACGGGAGGCAGCAG) and 806R (GGACTACHVGGGTWTCTAAT) [23] with TransStart Fastpfu DNA Polymerase (TransGen, China). The PCR program for target genes was optimized as follows: denaturation at 95 °C for 2 min; 20 cycles of amplification (45 s at 95 °C, 30 s at 55 °C, and 30 s at 72 °C); extension at 72 °C for 5 min [24]. The PCR products were detected through 2% agarose gel electrophoresis and subsequently purified using an AxyPrep DNA Gel Extraction Kit (AXYGEN, USA). The amplicons were then equally pooled and sequenced on an Illumina NovaSeq 6000 instrument (2 × 250 cycles) (Illumina, USA).

### Bioinformatics and statistical analysis

Raw FASTQ files were processed using QIIME2 (version 2024.2) [26]. Quality control of the raw sequences was performed with the DADA2 plugin in QIIME2 using the default parameters. The normalized Amplicon Sequence Variants (ASVs) from each sample were used to analyze microbiota richness, evenness, and diversity, including Abundance-based Coverage Estimator (ACE), Shannon evenness, and Shannon index.

Differences in bacterial composition among the four patient groups were assessed via permutational multivariate analysis of variance (PERMANOVA) using PAST software (version 4.16c) [25], with default parameters, based on Bray‒Curtis dissimilarity. Taxonomic classifications were performed using RDP Classifier (version 2.14) [26], with default parameters (80% threshold). Species identification was conducted using BLAST against the SILVA database (version 138.1) [27] and the HOMD database (version 15.23) [28], utilizing the highest score, identity scores > 97% and alignment scores > 97%.

Microbiota functional profiling was performed using PICRUSt2 and MetaCyc pathway annotation following 16S rRNA gene copy number normalization [29].

Microbiome Multivariable Associations with Linear Models (MaAsLin2) [30] was employed to analyze the differential abundance in microbial taxa and functional profiles, as well as to identify associations between microbiota features and clinical variables. The analysis was adjusted for potential confounding factors, including sex, age, and body mass index (BMI). For taxonomic analysis, the default significance threshold, adjusted *p* (*q*) < 0.25, and minimum relative abundance threshold, min_abundance = 0.0005, were considered statistically significant.

Coefficient relationships within species and associations between species and functional profiles were calculated using the Spearman correlation algorithm in an R package. Correlation parameters were set at coefficients > 0.35 or <-0.35 and adjusted *p* < 0.05.

## Results

### Participant characteristics

The participants were divided into four groups, as defined in the Methods (Supplementary Fig. S1 and Table 1). We collected a total of 122 stool samples and 112 throat swabs for this study. In the Control group, 62 stool samples and 58 throat swabs were collected from 62 patients (average age, 92.44 years). In the Re-infection group, 20 stool samples and 18 throat swabs were collected from 21 patients (average age, 93.33 years). In the Re-None group, 28 stool samples and 25 throat swabs were collected from 28 patients in the Re-None group (average age, 92.41 years). In the Infection group,12 stool samples and 11 throat swabs were collected from 13 patients (average age, 94 years).

In total, 32 patients completed all three samplings. However, only one patient in the Re-Infection group was in an active infection state at all three sampling timepoints. In contrast, five patients in the Re-None group and sixteen in the Control group remained infection-free across all three sampling timepoints. Notably, no patient in the Infection group tested positive at all three sampling points.

### Microbiota diversity across different groups

**Fig. 1:**
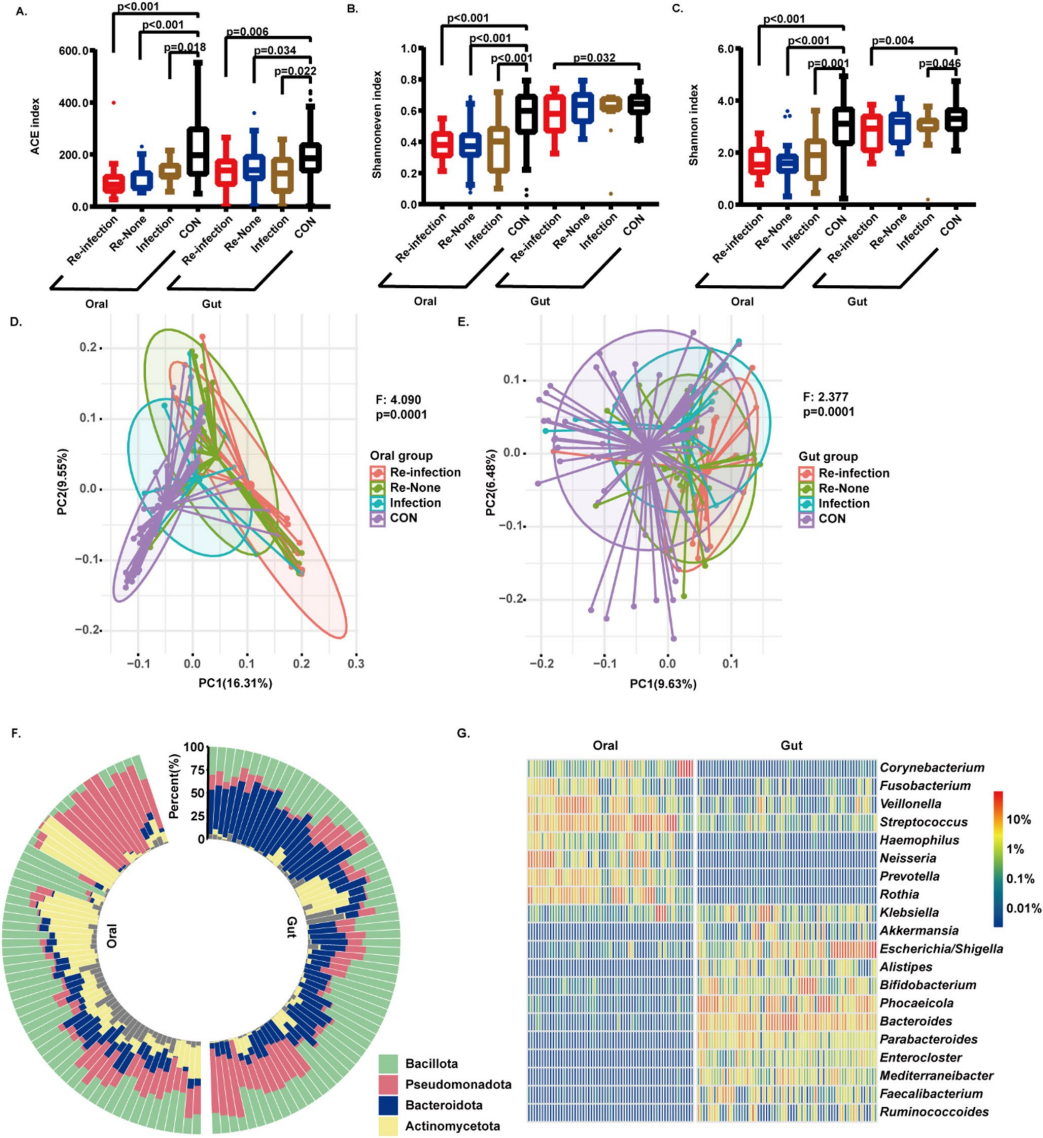
Diversity measures of microbial communities. (**A**) Richness index; (**B**) evenness index; (**C**) diversity index; (**D**) community structure shown by principal coordinate analysis (PCoA) of the oral microbiota; (**E**) community structure of the gut microbiota. (**F**) microbiome composition and phylum-level abundance of the control groups. (**G**) microbiome composition and genus-level abundance of the control groups. The *p* value and F value of the PCoA were calculated via PERMANOVA

To characterize microbial community variations, we conducted comprehensive alpha diversity analysis of oral and gut microbiota at the ASV level across all the groups. Three key indices were evaluated: ACE (richness), Shannon evenness, and Shannon diversity (Supplementary Table S1, Fig. 1). In the oral microbiota, the Control group exhibited significantly higher alpha diversity compared to the other groups, as indicated by elevated richness (ACE index, *p* < 0.05), evenness (Shannon evenness index, *p* < 0.05), and overall diversity (Shannon index, *p* < 0.05). A similar trend was observed in gut microbiota, though only the Control group showed significant increase in richness(*p* < 0.05) compared to the other groups. Notably, the Re-Infection group displayed significantly reduced alpha diversity indices for both oral and gut microbiota compared to the Control group (*p* < 0.05). However, comparative analysis among the Re-Infection, Re-None, and Infection groups revealed no significant differences in any of the three indices for either microbiota type (*p* > 0.05).

PERMANOVA revealed significant differences in beta diversity between the Control and test groups for both gut and oral microbiota (oral: F = 4.090, *p* < 0.05; gut: F = 2.377, *p* < 0.05). This finding was further supported by Principal Coordinate Analysis (PCoA), which revealed distinct clustering patterns between the Control and test groups (Fig. 1). Notably, the Re-Infection and Re-None groups exhibited similar microbiota structures (oral: F = 1.734, *p* > 0.05; gut: F = 1.063, *p* > 0.05). Significant differences were observed in the composition of both microbiota types between the Control and test groups (*p* > 0.05). Notably, oral microbiota exhibited more pronounced variations in the community structure than gut microbiota across all four groups.

### Common microbiota composition and abundance of the control group

To investigate the composition of microbial communities in the elderly population, the distributions of bacterial taxa at the phylum, family, and genus levels were calculated on data from the Control group. The results revealed that oral microbiota was dominated by four phyla, including *Bacillota* (average 31.99%), *Pseudomonadota* (26.59%), *Actinomycetota* (23.03%) and *Bacteroidota* (10.12%). The gut microbiota was dominated by three phyla (average > 10%, Fig. 1F), including *Bacillota* (average 36.74%), *Bacteroidota* (33.50%), and *Pseudomonadota* (19.60%).

At the genus level (Fig. 1G; Table 2), nine genera demonstrated relative abundances exceeding > 2% in the oral microbiota, whereas 12 showed similar predominance in the gut microbiota. *Klebsiella* was the only genus identified as highly abundant in both microbial niches.

**Table 2:** Abundant taxa of control group

Level	Feature		Oral								Gut					
			Re-Infection		Re-None		Infection		Control		Re-Infection		Re-None		Infection	Control
Genus	*Klebsiella*		45.87%		19.65%		12.49%		4.07%		9.10%		3.61%		2.46%	3.95%
Genus	*Corynebacterium*		15.75%		11.10%		10.80%		9.38%		0.83%		0.06%		0.05%	0.02%
Genus	*Streptococcus*		10.78%		21.22%		27.57%		16.21%		0.22%		0.32%		1.53%	0.84%
Genus	*Neisseria*		3.92%		2.75%		0.05%		7.76%		0.01%		0.00%		0.00%	0.00%
Genus	*Haemophilus*		0.66%		0.86%		0.60%		2.70%		0.01%		0.00%		0.00%	0.03%
Genus	*Veillonella*		0.59%		1.71%		5.17%		7.50%		0.67%		0.03%		0.36%	1.39%
Genus	*Prevotella*		0.56%		1.21%		0.69%		4.81%		0.00%		0.00%		0.00%	0.01%
Genus	*Rothia*		0.22%		3.20%		7.67%		9.89%		0.01%		0.02%		0.02%	0.02%
Genus	*Fusobacterium*		0.15%		1.79%		0.51%		2.59%		0.39%		0.02%		0.35%	0.40%
Genus	*Escherichia/Shigella*		0.03%		0.10%		0.02%		0.27%		3.97%		4.29%		6.87%	12.89%
Genus	*Bacteroides*		0.02%		0.27%		0.02%		0.04%		10.80%		10.59%		22.66%	12.49%
Genus	*Phocaeicola*		0.01%		0.06%		0.03%		0.05%		12.63%		7.18%		8.72%	12.69%
Genus	*Bifidobacterium*		0.01%		0.05%		0.05%		0.05%		18.96%		15.37%		3.89%	6.27%
Genus	*Parabacteroides*		0.01%		0.00%		0.00%		0.00%		5.63%		3.06%		12.45%	3.77%
Genus	*Ruminococcoides*		0.00%		0.00%		0.00%		0.00%		0.04%		0.02%		0.60%	2.15%
Genus	*Faecalibacterium*		0.00%		0.01%		0.01%		0.00%		0.86%		0.09%		5.42%	3.21%
Genus	*Enterocloster*		0.00%		0.00%		0.00%		0.00%		0.61%		1.24%		1.81%	2.23%
Genus	*Akkermansia*		0.00%		0.00%		0.00%		0.00%		4.96%		8.80%		2.42%	2.21%
Genus	*Mediterraneibacter*		0.00%		0.00%		0.00%		0.00%		2.22%		4.65%		0.83%	3.37%
Genus	*Alistipes*		0.00%		0.00%		0.00%		0.00%		3.12%		4.26%		1.67%	2.31%

### Difference in microbiota composition in patients with pneumonia

**Fig. 2:**
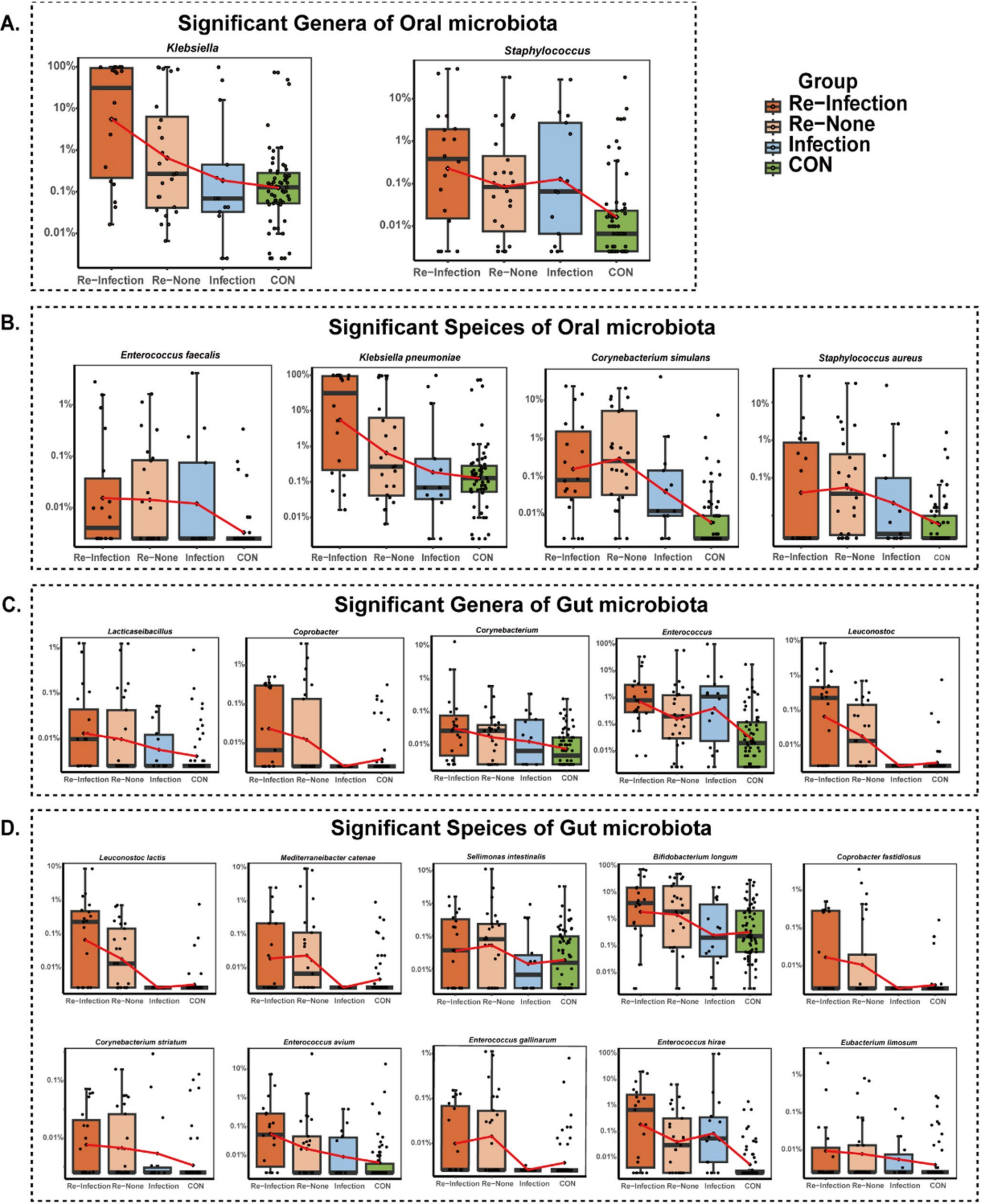
The microbiota which was significantly enriched in the Re-Infection and Re-None groups. Box plots showing different comparisons in oral or gut microbiota. In all the boxplots, the center line represents the median, the box limits represent upper and lower quartiles, whiskers represent minimum and maximum values, and the scattered points represent the values of each sample. (**A**) Genus level comparison in oral microbiota; (**B**) species level comparison in oral microbiota; (**C**) genus level comparison in gut microbiota; (**D**) species level comparison in gut microbiota. The ordinate is represented by the LOG percentage

To investigate microbial community shift associated with pneumonia, we compared the genus and species changes in the test group with those in the Control group adjusted for sex, age, and BMI by using MaAsLin2. The genus and species with significant changes (*q* < 0.25) are listed in Supplementary Table S2. In the oral microbiota, the abundances of 57 genera and 80 species showed significant changes in the test groups compared to those in the control group (Supplementary Table S2). Specifically, the abundances of 36 genera and 76 species decreased, those of two genera (*Klebsiella* and *Staphylococcus*) and four species (*Corynebacterium simulans*,* Klebsiella pneumoniae*,* Staphylococcus aureus* and *Enterococcus faecalis*) increased (Fig. 2). Seven genera abundant in the Control group were significantly different between the Re-Infection and Re-None groups. Notably, 33 genera and 65 species were commonly altered in the Re-Infection, Re-None, and Infection groups compared with those in the Control group. Only one genus, *Staphylococcus*, and three species, *C.simulans*, *E.faecalis*, and *S.aureus*, were significantly enriched in the Re-Infection, Re-None, and Infection three test groups, including the genera *Staphylococcus* and the species *C.simulans*, *E.faecalis*, and *S.aureus*. The oral microbiota of the Re-Infection and Re-None groups, which both experienced recurrent pneumonia infections, shared eight genera and 17 species whose abundances significantly differed from those of the Control group. The results (Fig. 2, Supplementary Table S2) indicate that the two genera and four species of oral microbiota that were associated with recurrent pneumonia.

In the gut microbiota, a total of 55 genera and 79 species were significantly altered in the test groups compared with those in the Control group (Table S2). At the genus level, three genera that were abundant in the Control group presented significant changes in the Re-Infection and Re-None groups (Table 2 and Supplementary Table S2). The gut microbiota of Re-Infection, Re-None, and Infection groups shared nine genera and 13 species compared with those of the Control group, and only one genus, *Enterococcus*, and one species, *Enterococcus hirae*, were significantly enriched in the Re-Infection, Re-None and Infection group (Fig. 2). The gut microbiota of the Re-Infection and Re-None groups presented 18 genera and 30 species whose abundances significantly differed from those of the Control group. Meanwhile, the abundances of four genera *Coprobacter*, *Corynebacterium*, *Lacticaseibacillus*, *Leuconostoc*, and nine species (*Leuconostoc lactis*,* Coprobacter fastidiosus*,* Bifidobacterium longum*,* Mediterraneibacter catenae*,* Corynebacterium striatum*,* Enterococcus avium*,* Sellimonas intestinalis*,* Enterococcus gallinarum* and *Eubacterium limosum*), were significantly greater in the test groups than those in the Control group. Thus, a total of five genera and ten species in the gut microbiota that were highly enriched in the Re-Infection and Re-None groups were associated with recurrent pneumonia.

Collectively, these findings suggest that the differences in oral microbiota are greater than those in the gut microbiota, and the highly enriched genera and species in the oral microbiota play a critical role in recurrent pneumonia.

### Differences between patients with and without recurrent pneumonia

We investigated the difference in microbiota between patients with or without recurrent pneumonia using MaAsLin2. The genera and species with significant changes (*q* < 0.25) are presented in Supplementary Table S3 (oral microbiota) and Supplementary Table S4 (gut microbiota). In terms of oral microbiota (Supplementary Table S3), a total of 8 genera and 11 species in the Re-Infection group were significantly different (*q* < 0.25) from those in the Infection group, with most of them being enriched in the Infection group.

Seven genera that were abundant in the Infection group, including *Limosilactobacillus*,* Streptococcus*,* Ligilactobacillus*,* Veillonella*, and *Lacticaseibacillus*, presented significant changes in the Re-Infection group. At the species level, 10 species that were abundant in the Infection group, including *Lacticaseibacillus casei*, *Veillonella tobetsuensis*, *Streptococcus ilei*, *Ligilactobacillus salivarius*, *Limosilactobacillus fermentum*, were significantly altered in the Re-Infection group. Notably, *K. pneumoniae* was differentially abundant across all test groups, with particularly high enrichment in the Re-Infection group (45.87%) and low abundance, and in the Infection group (12.49%) (Fig. 2, Supplementary Table S3. In comparison, the abundance of *K. pneumoniae* in the Control and Re-None groups was 4.07% and 19.65%, respectively.

In terms of the gut microbiota (Supplementary Table S4), a total of 8 genera and 14 species were significantly different between the Re-Infection and the Infection groups (*q* < 0.25). Five genera were enriched in the Re-Infection group, while three were enriched in the Infection group. Furthermore, seven species were enriched in the Re-Infection group, including *L. lactis*,* C. fastidiosus*,* Mediterraneibacter catenae*,* B. longum*, *E. gallinarum*,* Phocaeicola dorei* and *E. avium.* Among them, *B. longum* had an abundance of 15.16% in the Re-Infection group and 2.61% in the Infection group.

Altogether, these findings indicate that the Re-Infection groups exhibited greater enrichment of microbiota abundance than the Infection group, regardless of whether the focus was on oral or gut infections. Notably, *K. pneumoniae* demonstrated significantly higher relative abundance in the oral microbiota of the Re-infection group (*p* < 0.05).

### Species correlation and functional profiling

To reveal the intricate microbial interactions within the oral and gut microbiota, we analyzed the correlation patterns between enriched species and their counterparts. Among the four enriched species in the oral microbiota, *E. faecalis* and *S. aureus* were positively related to *C. simulans*, whereas *K. pneumoniae* was not related to any species. In in the gut microbiota, five of the 10 enriched species, including *B. longum*, *C. striatum*, *E. hirae*, *C. fastidiosus*, and *L. lactis*, showed no significant correlations with any other species. These results indicate that most enriched species are not strongly associated with other taxa.

Functional profiling analysis identified 420 pathways; their association with abundant species are presented in Supplementary Table S5 (*q* < 0.05). A total of 36 pathways showed significant associations with three oral-abundant species, while 29 pathways were significantly associated with four gut abundant species. In the oral microbiota, 23 pathways were positively correlated with the species *K. pneumoniae*, whereas 12 pathways were negatively correlated. Among the 23 positively related pathways, five were significantly enriched in the Re-Infection and Re-None groups compared with the Control group, all of which were involved in the degradation processes (Supplementary Fig. S2), such as GLUCARDEG-PWY (D-glucarate degradation I) and GALACTARDEG-PWY (D-galactarate degradation I). Among the 12 negatively related pathways, seven, including DENITRIFICATION-PWY (nitrate reduction I) and PWY-6470 (peptidoglycan biosynthesis V (beta-lactam resistance)), were enriched in the Re-Infection and Re-None groups compared with those in the Control group.

**Fig. 3:**
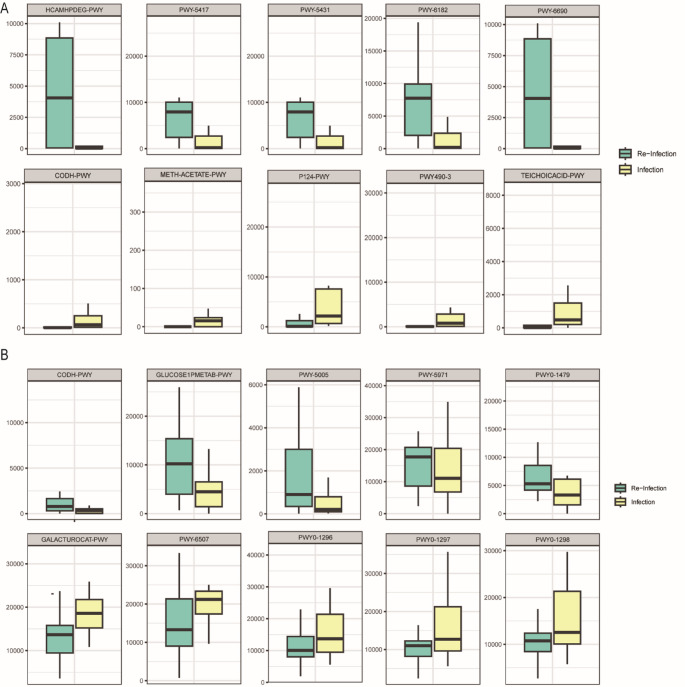
Differentially enriched pathways between the reinfected and infected groups. Box plots showing the Re-Infection group and the Infection group in different enriched pathways. In all the boxplots, the center line represents the median, the box limits represent upper and lower quartiles, and whiskers represent minimum and maximum values. (**A**) Pathways in the oral microbiota; (**B**) pathways in the gut microbiota

The functional differences in the microbiota between the Re-Infection and Infection groups were compared using MaAsLin2, and pathways with significant difference (*q* < 0.25) were presented in Supplementary Table S6. In the oral microbiota (Fig. 3A), a total of 43 pathways, including PWY-6182 (superpathway of salicylate degradation) and HCAMHPDEG-PWY (3-phenylpropanoate and 3-(3-hydroxyphenyl) propanoate degradation to 2-hydroxypentadienoate), were significantly (*q* < 0.25) enriched in the Re-Infection group. In contrast, 65 pathways, including PWY490-3 (nitrate reduction VI (assimilatory)) and P124-PWY (*Bifidobacterium* shunt), were significantly enriched (*q* < 0.25) in the Infection group. In the gut microbiota (Fig. 3B), a total of 46 pathways including PWY-5006 (biotin biosynthesis II) were found to be significantly elevated (*q* < 0.25) in the Re-Infection group. Additionally, 12 pathways, including PWY-6507 (4-deoxy-L-threo-hex-4-enopyranuronate degradation) and PWY0-1296 (purine ribonucleosides degradation) were significantly enriched (*q* < 0.25) in the Infection group.

### Associations between species and clinical data

Next, we analyzed the associations between microbiota features (species and genus) and clinical parameters using MaAsLin2. The significant associations (*q* < 0.25) are presented in Supplementary Table S7. Among the oral microbiota, 38 genera and 78 species were found to be associated with 11 clinical parameters, including blood albumin, C-reactive protein, hemoglobin, leukocyte, lymphocyte count, lymphocyte rate, neutrophil count, neutrophil rate, neutrophil-lymphocyte ratio (NLR), procalcitonin, and serum amyloid protein. In the gut microbiota, 21 genera and 33 species were associated with nine types of clinical data. In the oral microbiota, four enriched species were associated with nine of clinical parameters, whereas seven enriched species in the gut microbiota were associated with seven parameters. Furthermore, three clinical parameters—C-reactive protein, neutrophil count, and leukocyte count—were positively associated with four enriched oral species (*K. pneumoniae*, *S. aureus*, *C. simulans*, and *E. faecalis*) and five enriched gut species (*C. fastidiosus*,* E. hirae*, *M. catenae*,* E. limosum*, and *L. lactis*) (Supplementary Fig. S3 and Supplementary Table S7). The neutrophil-lymphocyte ratio was positively associated with four enriched oral species (*K. pneumoniae*, *S. aureus*, *C. simulans*, and *E. faecalis*), whereas procalcitonin level and lymphocyte count were positively associated with two enriched gut species (*M. catenae* and *L. lactis*).

## Discussion

Pneumonia, a common inflammatory condition, affects the air sacs in one or both lungs [7]. A large number of elderly patients may become infected with pneumonia in hospitals, where it is often classified as hospital-acquired pneumonia (HAP). The high hospitalization and mortality rates of HAP place a heavy burden on both the patients and healthcare system. Following the criteria proposed by Elena Chiappini et al. [31], we adopted > 5 infections per year as the threshold for recurrent pneumonia due to age-related immune decline and complex comorbidities in elderly patients. The oral microbiota has a crucial relationship with the with lung homeostasis [5]. A previous study used sputum samples to perform bacterial culture, which was time-consuming and had limited detection range [32]. To overcome these limitations, we used the high-throughput sequencing to investigate the differences in oral and gut microbiota of elderly individuals. These individuals were grouped according to their history of recurrent pneumonia and current infection status. Compared with the three test groups, the Control group presented significant differences in both the oral and gut microbiota.

The alpha diversity index indicates the relative abundance of various microbiota species across space and time [33]. ACE, Shannon evenness, and Shannon indices are frequently used to measure alpha diversity. In our study, the Control group exhibited greater richness (*p* < 0.05) from the other groups in terms of oral microbiota. The alpha indices of both the oral and gut microbiota in the Re-Infection group were significantly lower than those in the Control group. PERMANOVA and PCoA of beta diversity revealed that the Control group differed significantly from the other three groups in terms of both gut and oral microbiota. In addition, the Re-Infection and Re-None groups showed similarities in both microbiota types, suggesting that patients with recurrent lung infections possess similar microbiota structures in both the oral cavity and gut.

We investigated the composition of oral microbial communities at different levels, including phylum, family, and genus, to analyze the differences between the Control and test groups. MaAsLin2 was uesd for its specialization in identify multivariable associations within microbiome data [30], setting an FDR threshold of *q* < 0.25 to balance detection efficiency and false negatives given our modest sample size and multiple covariates (age, gender, and BMI). As shown in Supplementary Table S2, the oral microbiota of test groups patients was significantly altered (*q* < 0.25) compared with that of the Control group. The majority of the taxa were significantly decreased in the oral microbiota of Re-Infection, Re-None, and Infection groups patients. In addition, only one genus, *Staphylococcus*, and three species (*C. simulans*, *E. faecalis*, and *S. aureus*), were significantly higher in the Re-Infection, Re-None, and Infection groups (Fig. 2). *C. simulans*, a Gram-positive bacterium, has been reported as a causative pathogen of pneumonia, capable of impairing airway clearance and damaging airway structures [34]. *C. striatum* has also been reported to be increase in patient with HAP from 2014-2015 to 2018–2019 in South Korea [35]. *E. faecalis*, a Gram-positive *Enterococcus* is part of the normal gut microbiota [36]. However, it can cause a variety of infections, such as infective endocarditis, urinary tract infections, bacteremia, peritonitis, prosthetic joint infections, and endophthalmitis [36, 37]. *S. aureus* is another common opportunistic pathogen associated with a wide range of infections in the respiratory system, such as asymptomatic colonization, cystic fibrosis lung disease, and fulminant necrotizing pneumonia [7, 38]. Numerous studies have highlighted the association between *S. aureus* and HAP [39]. In fact, some *S. aureus* strains have developed resistance to antibiotics because of overuse which makes them more toxic and difficult to treat.

Compared with the Control group, the Re-Infection and Re-None groups exhibited a significantly higher abundance of one species: *K. pneumoniae*, a pathogenic bacteria associated with HAP [7]. In humans, *Klebsiella* frequently resides in the nasal and digestive tracts, typically in an asymptomatic colonization [40]. When the host immunity is weakened, *K. pneumoniae* may cause infections. The outer membrane of *K. pneumoniae* contains lipopolysaccharides and capsule polysaccharides, which help it escape the host immune system [10]. Infections caused by *K. pneumoniae*, particularly pneumonia, are associated with high mortality and are influenced by several risk factors, including virulence factors, antibiotic resistance, host genetics, age, immune status, antibiotic use, environmental exposure, and nutrition [40]. Our research confirmed that HAP cases in this study were correlated with *S. aureus* and *K. pneumoniae.* Importantly, t *K. pneumoniae* was more abundant in the Re-Infection group than in the Infection group within the oral microbiota. This suggests that it may be realted to the recurrent infections experienced by these patient groups. This enrichment pattern underscores the potential role of *K. pneumoniae* in increasing patient susceptibility to recurrent infections, warranting further investigation into its influence on oral microbiota stability.

Patients in the test groups also displayed some differences in their gut microbiota compared with those in the Control group (Fig. 2). Only one species, *E. hirae*, showed higher abundance in the Re-Infection, Re-None, and Infection groups than that in the Control group. However, studies on the relationship between *E. hirae* and pulmonary infection are scarce.

Furthermore, in the Re-Infection and Re-None groups, four genera and nine species were more abundant than they were in the Control group. Among these, both *B. longum* and *C. simulans* are related to pneumonia. *B. longum* acts as an immunomodulator and immunostimulant [41]. Groeger et al. confirmed that exposure to *B. longum* can protect against inflammatory sequelae and damage in a murine model of lethal influenza infection via intranasal administration [42]. Khailova et al. demonstrated that *Lactobacillus rhamnosus* GG and *B. longum* can attenuate lung injury and inflammatory response to experimental sepsis in a weanling mouse model of cecal ligation and puncture [43]. The abundance of these species in our study can be attributed to the patients in both the Re-Infection and Re-None groups having a prior history of pneumonia and subsequent recovery. As a result, their gut microbiota might have undergone alterations following the initial infection. This outcome merits close scrutiny and consideration. The enrichment of *B. longum* in the Re-infection group rather than in the Infection group also corroborates this conclusion.

Next, we studied the intricate microbial interactions within the oral and gut microbiota (Supplementary Table S2, *q* < 0.05). *E. faecalis* and *S. aureus* were found to be positively correlated to *C. simulans* in the oral microbiota. All three species were related to pneumonia infection and coexisted in the three test groups within the oral microbiota. Chudáček et al. investigated HAP in patients with secondary peritonitis and reported that the infection was introduced in these patients by pathogenic bacteria including *E. faecalis*,* S. aureus*, and *K. pneumoniae* [44]. Conversely, the investigation of the gut microbiota indicated that most enriched species had no significant relationships with other species. The reason for this lack of association may be that the gut microbiota becomes unstable and loses its diversity in elderly individuals [45]. Thus, the gut microbiota can be influenced by many factors, such as the age, environment, diet, and sexuality.

We predicted the functional profiles of enriched species in the oral and gut microbiota. Pathways enriched in the Re-Infection and Re-None groups improved the degradation of D-glucarate and D-galactarate (Supplementary Fig. S2). Thus, upregulating the associated D-glucarate degradation (GLUCARDEG-PWY) and D-galactarate degradation (GALACTARDEG-PWY) pathways can provide a better environment for microbial growth [46, 47]. We predicted that the initial infection and self-regulation employed by patients enhance degradation, potentially resulting in a more favorable environment for the microbiota. Seven pathways, including the DENITRIFICATION-PWY (nitrate reduction I) pathway was downregulated in both the Re-Infection and Re-None groups. Oral microbiota typically reduces nitrate to nitrite, which may be further reduced to nitric oxide (NO) in the body, affecting blood pressure, vascular function, and oxygen consumption [48]. Similarly, nitrate reduction is also important in the gut microbiota. acting as the first line of defense at high concentrations against ingested pathogens [48]. Diminished capacity for nitrate reduction may reduce NO levels, potentially allowing a greater number of pathogens to enter the gastrointestinal tract of individuals. NO can inhibit bacterial growth by reacting with bacterial DNA and blocking damage repair [49].

Next, we compared the functional differences in oral microbiota between the Re-infection group and Infection groups (Fig. 3A). The PWY-6182 pathway, representing the superpathway of salicylate degradation, was enriched in the Re-infection group. Domenico et al. suggested that sodium salicylate can reduce the production of capsular polysaccharide, which is the most important virulence factor of *K. pneumoniae* [50]. Thus, the increased activity of salicylate degradation in *K. pneumoniae* could account for its enhanced toxicity and increased likelihood of causing pulmonary infections. In the gut microbiota, the PWY-5005 pathway, known as biotin biosynthesis II, was enriched in the Re-infection group compared with the Infection group. Biotin (vitamin H or B7) serves as an enzyme cofactor essential for the metabolic fixation of carbon dioxide for bacterial survival [51]. Carfrae et al. modeled the human environment in mice and suggested that biotin biosynthesis is crucial during infection with *K. pneumoniae* [52]. A previous study reported that *K. pneumoniae* may infect the host via BioC methyltransferase [53]. Biotin is also vital for the growth and gene expression of *S. aureus.* Several studies have demonstrated that the biotin protein ligase is a potential target for combating drug-resistant pathogens [54–56]. Thus, biosynthesis pathways play a key role in the diagnosis and treatment of elderly individuals experiencing recurrent pneumonia.

Finally, we analyzed the associations between microbiota features and clinical data (Supplementary Fig. S3 and Supplementary Table S7) and certain positive correlations between them. The clinical data mentioned in this article are related to pneumonia and inflammatory infections [57].

Lung microbiota and the gut-lung axis are crucial determinants of respiratory infection susceptibility, progression, and outcome [58]. Since the oral microbiota serves as a source for the lung microbiota, dysbiosis in both the oral and gut microbiota can lead to respiratory diseases [59, 60]. The microbiota can protect against respiratory infection via granulocyte-macrophage colony-stimulating factor (GM-CSF) signaling., These include *Staphylococcus epidermidis* in the upper airway and *Lactobacillus reuteri*, *E. faecalis*, and *Lactobacillus crispatus* in the gut [61], Through Nod2-mediated recognition of microbial peptidoglycan, the microbiota primes a shared innate defense against Gram-negative and Gram-positive respiratory pathogens by promoting IL-17 A-dependent GM-CSF production. GM-CSF then translates the microbiota-derived signals, enhancing bacterial clearance from the lungs, primarily through activation of alveolar macrophages via the ERK/ROS signaling pathways [61].

Oral microbiota is one of the primary sources of respiratory infections. A two-sample Mendelian randomization study in east Asian populations revealed, that *Prevotella*,* Actinomyces*,* Streptococcus*, and *Centipeda* in the oral microbiota were associated with pneumonia [62]. Specifically, *S. pneumoniae* was found to significantly aggravate the infection [62]. Additionally, in a study of patients diagnosed with bacterial pneumonia, more than half of the lower respiratory tract specimens tested positive for *Fusobacterium nucleatum* via real-time PCR [63]. The oral microbes in dental plaques, periodontal pockets or saliva could be inhaled into the lower respiratory tract to aggravate the pneumonia [64]. Sumi et al. has demonstrated the presence of certain pneumonia pathogens, such as *S. pneumoniae*, *Hemophilus influenzae*, and *K. pneumonia* in the dental plaques of elderly individuals [65]. Different lifestyles and diets, such as smoking, drinking alcohol, consuming spicy foods, and undergoing antibiotic treatments, can alter microbiota composition. Hence, variations in microbiota composition may be observed across different studies.

In this study, we investigated the oral and gut microbiota of elderly patients, including their composition, relationships, and functional pathways. However, our study has some limitations. First, the number of samples was small and the subjects may only represent the elderly population in Shanghai Fourth People’s Hospital affiliated with Tongji University. Future studies must include larger samples sizes and a wider the sampling range to increase the persuasiveness of the results. In our subsequent work, we aim to explore the relationships between the oral, lung, and gut microbiota to better understand the pathogenesis of pneumonia infection.

## Conclusion

Pneumonia remains a significant clinical challenge among elderly hospitalized patients, highlighting the need to investigate potential associations between microbial communities and infection susceptibility. Our study revealed significant differences in the diversity of oral and gut microbiota between patients with recurrent and common pneumonia, suggesting a potential link between microbial composition and infection risk. Notably, the oral microbiota differed more significantly than gut microbiota between patients with recurrent pneumonia and common pneumonia. Specifically, the abundance of *S. aureus* significantly increased in the oral microbiota of patients once they are infected with pneumonia while *K. pneumoniae* were significantly increased in patients with recurrent infections. In the gut microbiota, *E. hirae* was the only species that was significantly enriched in the all three test groups compared to the Control. Compared with the Infection group, the abundance of *K. pneumoniae* was considerably increased in the Re-Infection group. Moreover, the degradation of D-glucarate and D-galactarate was enhanced in patients who experienced recurrent infections, creating a more favorable environment for microbial growth. In contrast, the DENITRIFICATION-PWY pathway was reduced in the oral microbiota of these patients, indicating a diminished capacity for nitrate reduction. This may allow a greater number of pathogens to enter the gastrointestinal tract. Based on our functional predictions, biotin biosynthesis pathways were upregulated in the Re-Infection group, which could be key to diagnosing and treating elderly individuals with recurrent pneumonia.

Collectively, increased prevalence of *S. aureus* and *K. pneumoniae* in the oral microbiota provides important etiological insights into pneumonia pathogenesis. Specifically, the enrichment of *K. pneumoniae* may explain the heightened susceptibility to pulmonary infections observed in the elderly population.

## Electronic supplementary material

Below is the link to the electronic supplementary material.

Supplementary Material 1

Supplementary Material 2

## Data Availability

The sequencing data from this study have been deposited in the National Omics Data Encyclopedia (NODE, https://www.biosino.org/node/index) under accession number OEX00029312 (oral microbiota) and OEX00029313 (gut microbiota), respectively.

## References

[1] Häder A, Köse-Vogel N, Schulz L et al (2023) Respiratory infections in the aging lung: implications for diagnosis, therapy, and prevention. Aging Dis 14:1091–1104. 10.14336/AD.2023.0329 37163442 10.14336/AD.2023.0329 PMC10389836

[2] Brescia B (2010) Recurrent respiratory infections in elderly. BMC Geriatr 10:L20. 10.1186/1471-2318-10-S1-L20

[3] Cunha BA (2001) Pneumonia in the elderly. Clin Microbiol Infec 7:581–588. 10.1046/j.1198-743x.2001.00328.x 11737082 10.1046/j.1198-743x.2001.00328.x

[4] Henig O, Kaye KS (2017) Bacterial pneumonia in older adults. Infect Dis Clin North Am 31:689–713. 10.1016/j.idc.2017.07.015 28916385 10.1016/j.idc.2017.07.015 PMC7127502

[5] Liu S, Xie G, Chen M et al (2023) Oral microbial dysbiosis in patients with periodontitis and chronic obstructive pulmonary disease. Front Cell Infect Microbiol 13:1121399. 10.3389/fcimb.2023.1121399 36844402 10.3389/fcimb.2023.1121399 PMC9948037

[6] Baker JL, Mark Welch JL, Kauffman KM et al (2024) The oral microbiome: diversity, biogeography and human health. Nat Rev Microbiol 22:89–104. 10.1038/s41579-023-00963-6 37700024 10.1038/s41579-023-00963-6 PMC11084736

[7] Dong J, Li W, Wang Q et al (2021) Relationships between oral microecosystem and respiratory diseases. Front Mol Biosci 8:718222. 10.3389/fmolb.2021.718222 35071321 10.3389/fmolb.2021.718222 PMC8767498

[8] Vieira AT, Rocha VM, Tavares L et al (2016) Control of klebsiella pneumoniae pulmonary infection and Immunomodulation by oral treatment with the commensal probiotic bifidobacterium longum 51A. Microbes Infect 18:180–189. 10.1016/j.micinf.2015.10.008 26548605 10.1016/j.micinf.2015.10.008

[9] Abbas R, Chakkour M, El Zein H et al (2024) General overview of klebsiella pneumonia: epidemiology and the role of siderophores in its pathogenicity. Biology 13:78. 10.3390/biology13020078 38392297 10.3390/biology13020078 PMC10886558

[10] Opoku-Temeng C, Kobayashi SD, DeLeo FR (2019) Klebsiella pneumoniae capsule polysaccharide as a target for therapeutics and vaccines. Comput Struct Biotechnol J 17:1360–1366. 10.1016/j.csbj.2019.09.011 31762959 10.1016/j.csbj.2019.09.011 PMC6861629

[11] Agyeman WY, Bisht A, Gopinath A et al (2022) A systematic review of antibiotic resistance trends and treatment options for hospital-acquired multidrug-resistant infections. Cureus 14:e29956. 10.7759/cureus.29956 36381838 10.7759/cureus.29956 PMC9635809

[12] Cheung GYC, Bae JS, Otto M (2021) Pathogenicity and virulence of staphylococcus aureus. Virulence 12:547–569. 10.1080/21505594.2021.1878688 33522395 10.1080/21505594.2021.1878688 PMC7872022

[13] Tong SYC, Davis JS, Eichenberger E et al (2015) Staphylococcus aureus infections: epidemiology, pathophysiology, clinical manifestations, and management. Clin Microbiol Rev 28:603–661. 10.1128/CMR.00134-14 26016486 10.1128/CMR.00134-14 PMC4451395

[14] Chen H, Zhang J, He Y et al (2022) Exploring the role of staphylococcus aureus in inflammatory diseases. Toxins 14:464. 10.3390/toxins14070464 35878202 10.3390/toxins14070464 PMC9318596

[15] Adak A, Khan MR (2019) An insight into gut microbiota and its functionalities. Cell Mol Life Sci 76:473–493. 10.1007/s00018-018-2943-4 30317530 10.1007/s00018-018-2943-4 PMC11105460

[16] Góralczyk-Bińkowska A, Szmajda-Krygier D, Kozłowska E (2022) The microbiota-gut-brain axis in psychiatric disorders. Int J Mol Sci 23:11245. 10.3390/ijms231911245 36232548 10.3390/ijms231911245 PMC9570195

[17] Kamada N, Chen GY, Inohara N, Núñez G (2013) Control of pathogens and pathobionts by the gut microbiota. Nat Immunol 14:685–690. 10.1038/ni.2608 23778796 10.1038/ni.2608 PMC4083503

[18] Ma P-J, Wang M-M, Wang Y (2022) Gut microbiota: A new insight into lung diseases. Biomed Pharmacother 155:113810. 10.1016/j.biopha.2022.113810 36271581 10.1016/j.biopha.2022.113810

[19] Rastogi S, Mohanty S, Sharma S, Tripathi P (2022) Possible role of gut microbes and host’s immune response in gut-lung homeostasis. Front Immunol 13:954339. 10.3389/fimmu.2022.954339 36275735 10.3389/fimmu.2022.954339 PMC9581402

[20] Wang S, Yang L, Hu H et al (2022) Characteristic gut microbiota and metabolic changes in patients with pulmonary tuberculosis. Microb Biotechnol 15:262–275. 10.1111/1751-7915.13761 33599402 10.1111/1751-7915.13761 PMC8719804

[21] Hiergeist A, Gläsner J, Reischl U, Gessner A (2015) Analyses of intestinal microbiota: culture versus sequencing. ILAR J 56:228–240. 10.1093/ilar/ilv017 26323632 10.1093/ilar/ilv017

[22] Krivonos DV, Fedorov DE, Konanov DN et al (2025) Pike: OTU-level analysis for Oxford nanopore amplicon metagenomics. Int J Mol Sci 26:4168. 10.3390/ijms26094168 40362406 10.3390/ijms26094168 PMC12071631

[23] Klindworth A, Pruesse E, Schweer T et al (2013) Evaluation of general 16S ribosomal RNA gene PCR primers for classical and next-generation sequencing-based diversity studies. Nucleic Acids Res 41:e1. 10.1093/nar/gks808 22933715 10.1093/nar/gks808 PMC3592464

[24] Li N, Wang Y, You C et al (2018) Variation in Raw milk microbiota throughout 12 months and the impact of weather conditions. Sci Rep 8:2371. 10.1038/s41598-018-20862-8 29402950 10.1038/s41598-018-20862-8 PMC5799204

[25] Hammer O, Harper DAT, Ryan PD PAST: paleontological statistics software package for education and data analysis

[26] Wang Q, Garrity GM, Tiedje JM, Cole JR (2007) Naïve bayesian classifier for rapid assignment of rRNA sequences into the new bacterial taxonomy. Appl Environ Microbiol 73:5261–5267. 10.1128/AEM.00062-07 17586664 10.1128/AEM.00062-07 PMC1950982

[27] Quast C, Pruesse E, Yilmaz P et al (2012) The SILVA ribosomal RNA gene database project: improved data processing and web-based tools. Nucleic Acids Res 41:D590–D596. 10.1093/nar/gks1219 23193283 10.1093/nar/gks1219 PMC3531112

[28] Chen T, Yu W-H, Izard J et al (2010) The human oral Microbiome database: a web accessible resource for investigating oral microbe taxonomic and genomic information. Database (Oxford) 2010(baq013). 10.1093/database/baq013 10.1093/database/baq013 PMC2911848 20624719

[29] Douglas GM, Maffei VJ, Zaneveld JR et al (2020) PICRUSt2 for prediction of metagenome functions. Nat Biotechnol 38:685–688. 10.1038/s41587-020-0548-6 32483366 10.1038/s41587-020-0548-6 PMC7365738

[30] Mallick H, Rahnavard A, McIver LJ et al (2021) Multivariable association discovery in population-scale meta-omics studies. PLoS Comput Biol 17:e1009442. 10.1371/journal.pcbi.1009442 34784344 10.1371/journal.pcbi.1009442 PMC8714082

[31] Chiappini E, Santamaria F, Marseglia GL et al (2021) Prevention of recurrent respiratory infections: inter-society consensus. Ital J Pediatr 47:211. 10.1186/s13052-021-01150-0 34696778 10.1186/s13052-021-01150-0 PMC8543868

[32] Zhong Q, Feng H, Lu Q et al (2018) Recurrent wheezing in neonatal pneumonia is associated with combined infection with respiratory syncytial virus and staphylococcus aureus or klebsiella pneumoniae. Sci Rep 8:995. 10.1038/s41598-018-19386-y 29343795 10.1038/s41598-018-19386-y PMC5772642

[33] Avalos-Fernandez M, Alin T, Métayer C et al (2022) The respiratory microbiota alpha-diversity in chronic lung diseases: first systematic review and meta-analysis. Respir Res 23:214. 10.1186/s12931-022-02132-4 35999634 10.1186/s12931-022-02132-4 PMC9396807

[34] Yatera K, Mukae H (2020) Corynebacterium species as one of the major causative pathogens of bacterial pneumonia. Respiratory Invest 58:131–133. 10.1016/j.resinv.2020.01.008 10.1016/j.resinv.2020.01.008 32184071

[35] Lee YW, Huh JW, Hong S-B et al (2022) Severe pneumonia caused by corynebacterium striatum in adults, seoul, South korea, 2014–2019. Emerg Infect Dis 28:2147–2154. 10.3201/eid2811.220273 36287034 10.3201/eid2811.220273 PMC9622248

[36] Brown AO, Singh KV, Cruz MR et al (2021) Cardiac microlesions form during severe bacteremic enterococcus faecalis infection. J Infect Dis 223:508–516. 10.1093/infdis/jiaa371 32597945 10.1093/infdis/jiaa371 PMC7881331

[37] Zhou Y, Zhou Z, Zheng L et al (2023) Urinary tract infections caused by uropathogenic escherichia coli: mechanisms of infection and treatment options. Int J Mol Sci 24:10537. 10.3390/ijms241310537 37445714 10.3390/ijms241310537 PMC10341809

[38] Parker D, Prince A (2012) Immunopathogenesis of staphylococcus aureus pulmonary infection. Semin Immunopathol 34:281–297. 10.1007/s00281-011-0291-7 22037948 10.1007/s00281-011-0291-7 PMC3577067

[39] Yin Y, Hountras P, Wunderink RG (2017) The Microbiome in mechanically ventilated patients. Curr Opin Infect Dis 30:208–213. 10.1097/QCO.0000000000000352 28067677 10.1097/QCO.0000000000000352

[40] Chang D, Sharma L, Dela Cruz CS, Zhang D (2021) Clinical epidemiology, risk factors, and control strategies of klebsiella pneumoniae infection. Front Microbiol 12:750662. 10.3389/fmicb.2021.750662 34992583 10.3389/fmicb.2021.750662 PMC8724557

[41] Niu X, Yin X, Wu X et al (2023) Heat-killed bifidobacterium longum BBMN68 in pasteurized yogurt alleviates mugwort pollen-induced allergic airway responses through gut microbiota modulation in a murine model. Foods (Basel Switz) 122049. 10.3390/foods12102049 10.3390/foods12102049 PMC10217734 37238867

[42] Groeger D, Schiavi E, Grant R et al (2020) Intranasal bifidobacterium longum protects against viral-induced lung inflammation and injury in a murine model of lethal influenza infection. EBioMedicine 60:102981. 10.1016/j.ebiom.2020.102981 32927273 10.1016/j.ebiom.2020.102981 PMC7495089

[43] Khailova L, Petrie B, Baird CH et al (2014) Lactobacillus rhamnosus GG and bifidobacterium longum attenuate lung injury and inflammatory response in experimental sepsis. PLoS ONE 9:e97861. 10.1371/journal.pone.0097861 24830455 10.1371/journal.pone.0097861 PMC4022641

[44] Chudáček J, Špička P, Kolar M et al (2023) Analysis of bacterial pathogens causing complicating HAP in patients with secondary peritonitis. Antibiot (Basel Switz) 12:527. 10.3390/antibiotics12030527 10.3390/antibiotics12030527 PMC10044605 36978393

[45] Thibeault C, Suttorp N, Opitz B (2021) The microbiota in pneumonia: from protection to predisposition. Sci Transl Med 13:eaba0501. 10.1126/scitranslmed.aba0501 33441423 10.1126/scitranslmed.aba0501

[46] Liu N-H (2023) Fresh washed microbiota transplantation alters gut microbiota metabolites to ameliorate sleeping disorder symptom of autistic children. J Microbiol 61:741–753. 10.1007/s12275-023-00069-x 37665552 10.1007/s12275-023-00069-x

[47] Liu Y, Zeng Y, Liu Y et al (2022) Regulatory effect of isomaltodextrin on a high-fat diet mouse model with LPS-induced low-grade chronic inflammation. J Agric, Food Chem 10.1021/acs.jafc.2c03391 36041062

[48] González-Soltero R, Bailén M, de Lucas B et al (2020) Role of oral and gut microbiota in dietary nitrate metabolism and its impact on sports performance. Nutrients 12:3611. 10.3390/nu12123611 33255362 10.3390/nu12123611 PMC7760746

[49] Yuan Z, Lin C, He Y et al (2020) Near-Infrared Light-Triggered Nitric-Oxide-Enhanced photodynamic therapy and Low-Temperature photothermal therapy for biofilm elimination. ACS Nano 14:3546–3562. 10.1021/acsnano.9b09871 32069025 10.1021/acsnano.9b09871

[50] Domenico P, Schwartz S, Cunha BA (1989) Reduction of capsular polysaccharide production in Klebsiella pneumoniae by sodium salicylate. Infect Immun 57:3778–3782. 10.1128/iai.57.12.3778-3782.1989 2680983 10.1128/iai.57.12.3778-3782.1989 PMC259904

[51] Lin S, Cronan JE (2011) Closing in on complete pathways of biotin biosynthesis. Mol Biosyst 7:1811. 10.1039/c1mb05022b 21437340 10.1039/c1mb05022b

[52] Carfrae LA, MacNair CR, Brown CM et al (2019) Mimicking the human environment in mice reveals that inhibiting biotin biosynthesis is effective against antibiotic-resistant pathogens. Nat Microbiol 5:93–101. 10.1038/s41564-019-0595-2 31659298 10.1038/s41564-019-0595-2

[53] Su Z, Zhang W, Shi Y et al A bacterial methyltransferase that initiates biotin synthesis, an attractive anti-ESKAPE druggable pathway. Sci Adv 10:eadp3954. 10.1126/sciadv.adp3954 10.1126/sciadv.adp3954 PMC11661456 39705367

[54] Pendini NR, Yap MY, Traore Da. K, et al (2013) Structural characterization of Staphylococcus aureus biotin protein ligase and interaction partners: an antibiotic target. Protein Sci: Publ Protein Soc 22:762–773. 10.1002/pro.2262 10.1002/pro.2262 PMC3690716 23559560

[55] Satiaputra J, Shearwin KE, Booker GW, Polyak SW (2016) Mechanisms of biotin-regulated gene expression in microbes. Synth Syst Biotechnol 1:17–24. 10.1016/j.synbio.2016.01.005 29062923 10.1016/j.synbio.2016.01.005 PMC5640590

[56] Soares da Costa TP, Tieu W, Yap MY et al (2012) Selective Inhibition of biotin protein ligase from Staphylococcus aureus. J Biol Chem 287:17823–17832. 10.1074/jbc.M112.356576 22437830 10.1074/jbc.M112.356576 PMC3366794

[57] Karakioulaki M, Stolz D (2019) Biomarkers in pneumonia-beyond procalcitonin. Int J Mol Sci 20:2004. 10.3390/ijms20082004 31022834 10.3390/ijms20082004 PMC6514895

[58] Marrella V, Nicchiotti F, Cassani B (2024) Microbiota and immunity during respiratory infections: lung and gut affair. Int J Mol Sci 25:4051. 10.3390/ijms25074051 38612860 10.3390/ijms25074051 PMC11012346

[59] Liu X, Shi F, Zeng J et al (2025) Oral microbiota and respiratory diseases: advances and perspectives. Clin Microbiol Rev 0:e00150–e00124. 10.1128/cmr.00150-24 10.1128/cmr.00150-24 PMC12160517 40172191

[60] Hou K, Wu Z-X, Chen X-Y et al (2022) Microbiota in health and diseases. Signal Transduct Target Ther 7:135. 10.1038/s41392-022-00974-4 35461318 10.1038/s41392-022-00974-4 PMC9034083

[61] Brown RL, Sequeira RP, Clarke TB (2017) The microbiota protects against respiratory infection via GM-CSF signaling. Nat Commun 8:1512. 10.1038/s41467-017-01803-x 29142211 10.1038/s41467-017-01803-x PMC5688119

[62] He J, Mao N, Lyu W et al (2024) Association between oral Microbiome and five types of respiratory infections: a two-sample Mendelian randomization study in East Asian population. Front Microbiol 15:1392473. 10.3389/fmicb.2024.1392473 38659993 10.3389/fmicb.2024.1392473 PMC11039966

[63] Nagaoka K, Yanagihara K, Harada Y et al (2017) Quantitative detection of periodontopathic bacteria in lower respiratory tract specimens by real-time PCR. J Infect Chemother 23:69–73. 10.1016/j.jiac.2016.09.013 27894820 10.1016/j.jiac.2016.09.013

[64] Shi T, Wang J, Dong J et al (2023) Periodontopathogens porphyromonas gingivalis and fusobacterium nucleatum and their roles in the progression of respiratory diseases. Pathog (basel Switz) 12:1110. 10.3390/pathogens12091110 10.3390/pathogens12091110 PMC10535846 37764918

[65] Sumi Y, Miura H, Michiwaki Y et al (2007) Colonization of dental plaque by respiratory pathogens in dependent elderly. Arch Gerontol Geriatr 44:119–124. 10.1016/j.archger.2006.04.004 16723159 10.1016/j.archger.2006.04.004

