# Supplementary material for: Pathogenic bacteria enriched in the oral microbiota might be associated with recurrent pulmonary infections in elderly individuals
Source: Aging Clin Exp Res. 2025 Aug 13;37(1):247. doi: 10.1007/s40520-025-03141-1 (PMC12350567; doi:10.1007/s40520-025-03141-1)
Supplement: Supplementary file 1 — Supplementary Material 1 [file 40520_2025_3141_MOESM1_ESM.docx]

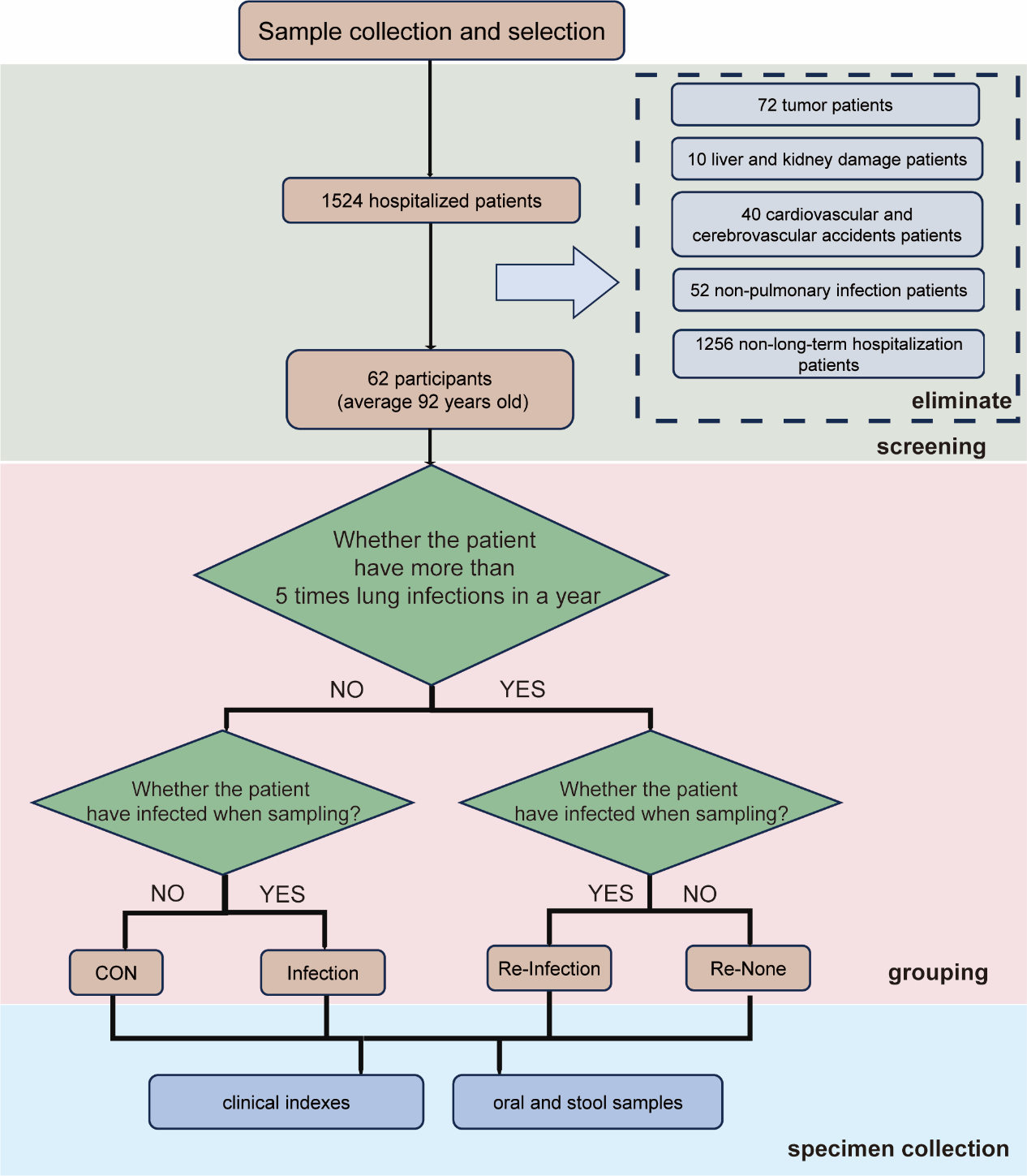


**Fig.S1 The workflow of sample collection**


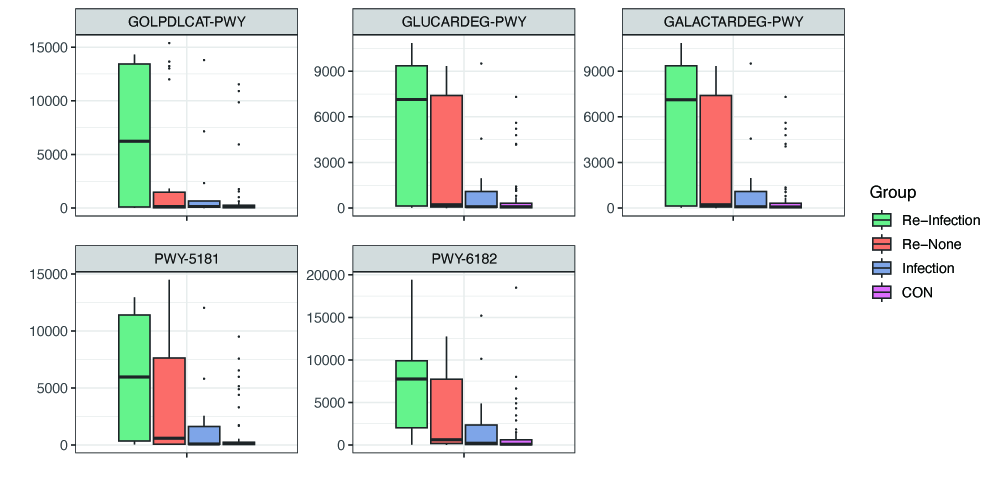


**Fig.S2** **The enriched pathways related to the species *Klebsiella pneumoniae* in the oral microbiota.**


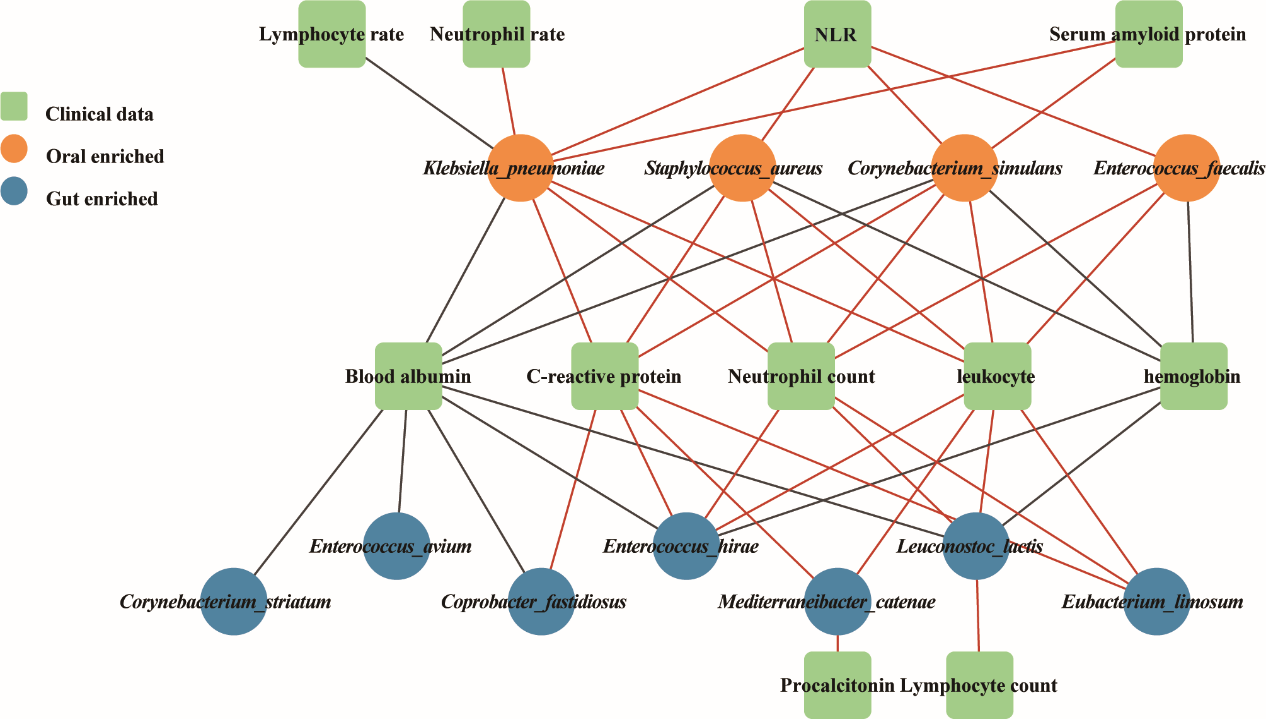


**Fig.S3 Associations between enriched species and clinical data.** The red line represents positive relationships, and the black line represents negative relationships.
